# The panorama of clinical trials for pediatric or adolescent sepsis: current status and future directions

**DOI:** 10.1097/JS9.0000000000002745

**Published:** 2025-06-12

**Authors:** De-Peng Lu, Lang-Lang Yang, Yu-Ting Huang, Xi Chen

**Affiliations:** a Department of Gastroenterology, First Affiliated Hospital of Anhui Medical University, Hefei, China; b Anhui Provincial Key Laboratory of Digestive Disease, First Affiliated Hospital of Anhui Medical University, Hefei, China

Sepsis remains a leading cause of morbidity and mortality in pediatric and adolescent populations, with an estimated 3.4 million annual deaths globally^[1]^. Despite advancements in critical care, therapeutic options remain limited, particularly for younger patients, due to physiological heterogeneity, ethical constraints, and a dearth of pediatric-specific drug development. Current guidelines rely heavily on extrapolation from adult studies, underscoring unmet clinical needs for age-tailored therapies. Analyzing the landscape of clinical trials in this domain is imperative to identify gaps, prioritize research, and accelerate translational progress. This Research Letter delineates the current status and future directions of clinical trials targeting pediatric or adolescent sepsis, emphasizing actionable insights for stakeholders.

We conducted a comprehensive search of Trialtrove database (https://clinicalintelligence.citeline.com/), an authoritative clinical trial database integrating clinical trial data from dozens of countries and regions around the world, including ClinicalTrials.gov and WHO registries, as of 17 April 2025 utilizing the retrieval strategy: “Patient Segment is Sepsis: Pediatric or Adolescent”. Analyzed metrics contained temporal trends of clinical trials, trial phases/status, mechanism of action (MOA), primary tested drugs, sponsor types, and geographic distribution. Two individual investigators independently reviewed and rechecked the source data to ensure the accuracy of the relevant conclusions: examples including extracting unstructured data, handling information gaps and classification bias to achieve relatively strict quality control. Any remaining limitations following the above quality control procedures do not affect the overall analysis trends of this study. And we ensure that our study is compliant with the TITAN Guidelines 2025^[2]^.

**HIGHLIGHTS**

- **Comprehensive landscape analysis**: The first systematic evaluation of clinical trials targeting pediatric/adolescent sepsis, offering evidence-based insights into trial phases, mechanisms of action, and geographic distribution.
- **Actionable recommendations**: Prioritizes strategies for integrating emerging therapies (e.g., immunomodulators, microbiome modulators) and digital solutions into clinical practice.
- **Global relevance**: Highlights disparities in trial representation and underscores opportunities for regional leadership in sepsis research.

A total of 615 eligible trials were identified eventually **(Table S1** http://links.lww.com/JS9/E391). Temporal analysis showed an increase of trial number from 1998 to 2023 (Fig. 1A). Phase 4 real-world studies dominated in the pipelines, followed by phase 2 trials (Fig. 1B). Most of the trials are completed, with 65 trials being open (Fig. 1C). Cell wall synthesis inhibitors constituted 32% of the trials, followed by drugs with unidentified pharmaceutical activity, lactamase inhibitor, microbiome modulators (defined as therapeutic interventions designed to intentionally alter the composition or function of the microbiome to achieve a clinical benefit^[3,4]^) and protein synthesis inhibitors (Fig. 1D). And undisclosed antibiotic drugs dominated in the primary tested drugs, emerging with probiotic therapies (Fig. 1D). For primary endpoints, mortality, safety and tolerability are the two most important indicators (Fig. 1E). With regard to sponsor types, academic institutions sponsored 77.9% of trials, versus 18.7% by industry (Fig. 1F; Table S1 http://links.lww.com/JS9/E391). Geographically, 19.2% of trials were conducted in the United States, followed by India, with limited representation from Italy and Japan (Fig. 1G), which partially aligns with the variations in national disease burden and healthcare systems.

**Figure 1.**
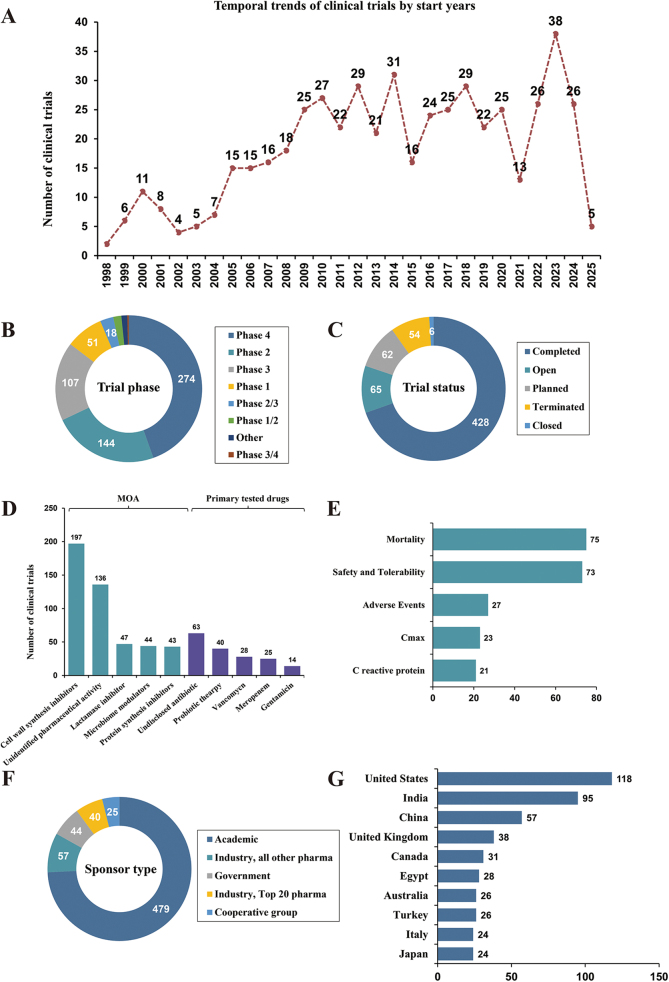
The panorama of clinical trials for pediatric or adolescent sepsis. **A.** Temporal trends of clinical trials by start years; **B.** trial phase distribution of clinical trials; **C.** Trial status distribution of clinical trials; **D.** Primary tested drugs and mechanisms of actions (Top 5); **E.** Primary endpoint distribution of clinical trials (Top 5); **F.** Sponsor type distribution of clinical trials (Top 5); **G.** Country distribution of clinical trials (Top 10).

Emphatically, the disclosed clinical trial outcomes demonstrated encouraging safety and efficacy data of emerging therapeutics in treating pediatric or adolescent sepsis. For instance, probiotics supplementation for 7 days resulted in significant decrease in proinflammatory and increase in anti-inflammatory cytokines in children with severe sepsis^[5]^. In addition, there is evidence indicating that in very low birth weight infants, probiotic (*Bifidobacterium lactis*) and synbiotic (*Bifidobacterium lactis* plus inulin) but not prebiotic (inulin) alone decrease necrotizing enterocolitis^[6]^. Despite potential undisclosed negative results, the current evidence collectively underscores the considerable therapeutic promise of microbiome modulators for pediatric and adolescent sepsis. Meanwhile, it is imperative to highlight that the updated international consensus criteria for pediatric sepsis and sepsis shock provide the up-to-date guidelines for future clinical practice^[7]^. Digital solutions in pediatric sepsis, likewise, injected fresh blood into this evolving field^[8]^. Also, preclinical advances, such as immunomodulators and microbiome-targeted therapies, offer novel therapeutic pathways^[9]^. In addition, emphatically, sepsis-associated multi-organ dysfunction syndrome (MODS) and hepatic injury, prevalent in pediatric cohorts, warrant targeted exploration^[10]^. Meanwhile, emerging biomarkers (e.g., cell-free DNA, glycocalyx degradation products) may refine patient selection.

In conclusion, this analysis underscores fragmented progress in pediatric sepsis drug development, marked by geographic disparities, sponsor reliance on academia, and a need for mechanistic diversification. Future efforts must prioritize global collaboration, adaptive trial designs, and integration of preclinical breakthroughs targeting MODS and organ-specific injury. Investment in precision medicine frameworks, coupled with regulatory incentives for pediatric sepsis research, will be pivotal to transforming this bleak panorama into actionable therapeutic victories.

## Data Availability

All data for this study have been provided.
